# Timing of Antenatal Corticosteroid Administration and Neonatal Outcomes

**DOI:** 10.1001/jamanetworkopen.2025.11315

**Published:** 2025-05-19

**Authors:** Nir Melamed, Kellie E. Murphy, Christy Pylypjuk, Rebecca Sherlock, Guillaume Ethier, Eugene W. Yoon, Prakesh S. Shah

**Affiliations:** 1 Division of Maternal-Fetal Medicine, Department of Obstetrics and Gynaecology, Sunnybrook Health Sciences Centre, Temerty Faculty of Medicine, University of Toronto, Toronto, Ontario, Canada; 2 Division of Maternal-Fetal Medicine, Department of Obstetrics and Gynaecology, Mount Sinai Hospital, Temerty Faculty of Medicine, University of Toronto, Toronto, Ontario, Canada; 3 Department Obstetrics, Gynecology and Reproductive Sciences, University of Manitoba, Winnipeg, Canada; 4 Department of Pediatrics, Surrey Memorial Hospital, University of British Columbia, Vancouver, Canada; 5 Department of Neonatology, CHU Sainte-Justine, Montreal, Quebec, Canada; 6 Maternal-Infant Care Research Centre (MiCare), Mount Sinai Hospital, Toronto, Ontario, Canada; 7 Department of Neonatology, Mount Sinai Hospital, Temerty Faculty of Medicine, University of Toronto, Toronto, Ontario, Canada

## Abstract

**Importance:**

Antenatal corticosteroids (ACS) are accepted to be most effective in reducing prematurity-related neonatal mortality and morbidity when administered 1 to 7 days before birth. However, precise data on the optimal timing of administration are scarce.

**Objective:**

To investigate the association between ACS administration to birth interval as a continuous variable and neonatal outcomes among preterm neonates.

**Design, Setting, and Participants:**

This national retrospective cohort study was conducted from 2018 to 2021 at level III neonatal intensive care units participating in the Canadian Neonatal Network. Participants included singleton and twin neonates born from 23 weeks 0 days’ to 31 weeks 6 days’ gestation. Data were analyzed from November 29, 2023, to March 8, 2024.

**Exposure:**

ACS administration to birth interval.

**Main Outcomes and Measures:**

The primary outcome was neonatal mortality. The secondary outcome was a composite of mortality or severe neurologic injury. Associations of the ACS administration to birth interval with the study outcomes were modeled using restricted cubic splines with 5 knots.

**Results:**

A total of 7950 neonates met the study criteria, from 7124 pregnancies (mean [SD] maternal age, 31.1 [5.7] years). Compared with individuals who received ACS, those who did not were younger and had lower rates of nulliparity, twin pregnancy, hypertension, and gestational diabetes. The overall rates of neonatal mortality and the composite outcome were 8% (670 of 7950) and 14% (1132 of 7950), respectively. ACS exposure was associated with reduced neonatal mortality as early as 2 hours after administration (adjusted risk ratio [ARR], 0.83 [95% CI, 0.70-1.00]). The reduction in mortality risk increased to a plateau 12 hours following exposure (ARR, 0.56 [95% CI, 0.40-0.78]), remained stable for the first 2 weeks following exposure, and gradually decreased after that. The association with reduced mortality was no longer observed at 4 weeks after administration (ARR, 0.82 [95% CI, 0.56-1.20]), and the ARR approached the null 5 weeks after administration (ARR, 0.99 [95% CI, 0.56-1.73]). This pattern was not affected by gestational age at birth or the number of fetuses.

**Conclusions and Relevance:**

In this cohort of neonates born from 23 weeks 0 days’ gestation to 31 weeks 6 days’ gestation, ACS administration was associated with a reduction in neonatal mortality as early as 2 hours after the administration of the first dose. The interval associated with the greatest reduction in neonatal mortality was between 12 hours and 14 days before birth, which was wider than the currently accepted optimal interval of 1 to 7 days. These findings may have important clinical implications for the management of pregnancies at risk of preterm birth, particularly regarding the administration of ACS even in cases in which preterm birth is imminent, and the timing of repeat courses of ACS.

## Key Points

**Question:**

Among preterm neonates exposed to antenatal corticosteroids (ACS), how quickly is an association observed between ACS administration and neonatal outcomes, and how long does the association persist?

**Findings:**

In this cohort study of 7950 neonates born between 23 weeks 0 days’ and 31 weeks 6 days’ gestation, ACS administration was associated with a reduction in neonatal mortality within 2 hours following administration. The optimal interval of ACS administration to birth was between 12 hours and 14 days.

**Meaning:**

These findings may support the administration of ACS even in cases in which preterm birth is imminent, and may affect the timing of repeat courses of ACS.

## Teaser

This cohort study examines the timing of antenatal corticosteroid administration as a continuous variable to assess the earliest, latest, and optimal times when exposure to corticosteroids before birth are associated with reduced mortality among preterm neonates.

## Introduction

The administration of a single course of antenatal corticosteroids (ACS) reduces neonatal morbidity and mortality and remains the single most effective intervention for pregnant individuals at risk of preterm birth before 34 weeks of gestation, primarily through the direct effects of ACS on fetal lung development.^1,2,3,4,5,6,7,8^ Accordingly, it is imperative that this intervention be used in a manner that maximizes its benefits.

The effects of ACS are influenced by the timing of its administration in relation to birth,^9,10,11^ a finding attributed to the transient effects of ACS on surfactant production by type 2 pneumocytes.^12,13,14^ While there is general agreement that ACS are most effective when administered within 1 to 7 days before birth,^7,10,11,15,16,17,18,19,20,21,22,23,24,25^ it remains unclear how soon within the first 24 hours ACS begin to take effect, when their maximal effect is achieved, and how long their benefits persist beyond the first 7 days. These questions, which define the beneficial interval of ACS (the period during which they exert any benefit) and the optimal interval of ACS (the period during which they exert their maximal benefit), are of major importance as they may determine whether ACS should be administered in cases in which preterm birth is imminent, determine the optimal timing of ACS administration, and guide the timing of a repeat (rescue) course of ACS.^16,26^ This knowledge gap arises because most published studies treated the interval between ACS administration and birth as a categorical variable, using a wide range of arbitrary, predetermined categories. A recent systematic review identified 65 different interval categories, concluding that this variation is a major barrier to synthesizing data and refining our understanding of the association between the timing of ACS administration and neonatal outcomes.^27^ The authors of the systematic review emphasized that advanced statistical modeling, where the interval is expressed as a continuous variable rather than arbitrary predetermined ranges, is essential to improve our understanding of this association, including the earliest onset and duration of the beneficial outcomes associated with this intervention.

Data on the association between the interval of ACS administration to birth as a continuous variable and neonatal outcomes are scarce and inconsistent. This inconsistency arises, in part, from methodological challenges, including residual confounding, insufficient sample size, variability in the study populations, reported outcomes, ACS regimens, and the choice of the reference group.^28,29^ Consequently, there is a pressing need for additional research to explore the association between the interval of ACS administration to birth as a continuous variable and neonatal outcomes to more precisely identify the beneficial and optimal intervals associated with ACS administration.^27^ In the current study, we aimed to investigate the association between the interval of ACS administration to birth as a continuous variable and neonatal mortality and morbidity in a large, national cohort of very preterm infants, with the goal of identifying the beneficial and optimal intervals of ACS administration.

## Methods

### Study Design and Population

We conducted a national retrospective cohort study of singleton and twin live neonates born between 23 weeks 0 days’ and 31 weeks 6 days’ gestation who were admitted to level III neonatal intensive care units (NICUs) participating in the Canadian Neonatal Network (CNN) and the Canadian Preterm Birth Network (CPTBN) between January 2018 and December 2021. The following cases were excluded: neonates from triplet and higher-order multiple gestations, neonates with major congenital anomalies, cases with unknown timing of administration of ACS, administration of more than 1 course of ACS, patients receiving chronic steroid treatment for preexisting conditions (such as autoimmune disease), and interval of ACS administration to birth of 8 or more weeks (as this implies ACS administration before 23 weeks’ gestation, which may represent a documentation error or administration of ACS outside protocol). The rationale for including twin pregnancies but not triplets or higher-order multifetal pregnancies is that, while evidence suggests that the benefit associated with ACS exposure in twins is comparable to that observed in singletons,^30^ similar data are unavailable for triplets and higher-order multifetal pregnancies. This study adhered to the Strengthening the Reporting of Observational Studies in Epidemiology (STROBE) reporting guideline for cohort studies. Data collection for the network was approved by the local research ethics boards or quality improvement committees. Additionally, we obtained approval from the Sunnybrook Health Sciences Centre Research Ethics Board for a retrospective investigation of this dataset and from the executive committee of the CPTBN. Informed consent was waived for this study because of the retrospective, population-based study design.

### Data Source

The CPTBN/CNN maintains a national database of outcomes, risk factors, and practices for infants admitted to level III NICUs across Canada. At each site, trained abstractors collect data from patient records using standardized definitions,^31^ which are then entered electronically into a customized data entry program with built-in error checks. The database has been shown to have high accuracy and internal consistency.^32^ During the study period, 31 NICUs participated in data collection for the CPTBN. We estimate that the database includes approximately 90% of the neonates born within this gestational age group in Canada. The lack of data on the remaining individuals is primarily attributable to the loss from the database of several neonates born at longer than 32 weeks’ gestation as they were born and cared for in level II units.

### Exposure

The exposure of interest was the ACS administration to birth interval, defined as the time from the administration of the first dose of ACS to birth. This exposure was considered as a continuous variable in units of hours, days, or weeks. The control (reference) group consisted of neonates not exposed to ACS. In addition, the ACS administration to birth interval was analyzed as a categorical variable based on the time from the administration of the first dose of ACS: (1) no ACS; (2) less than 24 hours; (3) 24 hours to 7 days; (4) 8 to 14 days; and (5) more than 14 days before birth. This categorical analysis was conducted to confirm the fidelity of the cohort using commonly studied predetermined intervals.

Exposure to ACS consisted of either betamethasone (administered intramuscularly in 2 doses of 12 mg 24 hours apart) or dexamethasone (administered intramuscularly in 4 doses of 6 mg 12 hours apart). While the CPTBN/CNN database does not include information on the specific agent used, it is likely that the majority of neonates were exposed to betamethasone, as it is the most commonly used type of ACS in Canada.

### Outcomes

The primary outcome was neonatal mortality before hospital discharge. The rationale underlying this choice is that this outcome represents an important clinical end point, is robust, and is less likely to be influenced by variations in local protocols or definitions.

We also included a secondary composite outcome of mortality or severe neurologic injury defined as severe (grade 3 or 4) intraventricular hemorrhage based on the criteria of Papile et al^33^ or periventricular leukomalacia. Due to advancements in postnatal respiratory care practices (such as early continuous positive airway pressure, prophylactic surfactant administration, and controlled oxygen use), the diagnosis of respiratory distress syndrome has become difficult to ascertain. Therefore, it was not included as an outcome in the current study.

### Covariables and Definitions

We considered covariables that could be potential confounding variables for the association between the ACS administration to birth interval and neonatal outcomes based on clinical knowledge and existing literature. The following variables were included: maternal age, plurality (twins vs singletons), hypertensive disorders of pregnancy (preeclampsia and gestational hypertension), birth weight below the 10th percentile for gestational age (based on a national reference,^34^ used as a surrogate for fetal growth restriction), and outborn admission (infants born in a non–level III center). Maternal race and ethnicity were considered potential confounders prior to the analysis; however, as the database we used did not collect information on these variables, we were unable to adjust them. Gestational age at the time of ACS administration and at birth was determined using the following sources, in order of priority: date of in vitro fertilization, first-trimester ultrasonography results, last menstrual period, obstetric estimate, and pediatric estimate.

### Statistical Analysis

Baseline characteristics were compared between the exposure groups using a 1-way analysis of variance and the χ^2^ test for continuous and categorical variables, respectively. The associations between the ACS administration to birth interval (as a continuous variable, expressed in hours) and the study outcomes were modeled using restricted cubic splines with 5 knots. Knot placement was determined using the Harrell method at the 5th, 27.5th, 50th, 72.5th, and 95th percentiles (1, 3, 6, 11, and 21 hours for analysis of the first 24 hours; 2, 10, 44, 104, and 269 hours for analysis of the first 14 days; and 2, 13, 64, 167, and 587 hours for analysis of the first 5 weeks, respectively).^35^ We used generalized linear models, assuming a Poisson distribution, to estimate the risk ratios (RRs) with 95% CIs using the nonexposed group as the reference. Twin pairs were considered as clusters to account for correlation within the pairs. Models were adjusted for the aforementioned covariables and for gestational age at birth. The analysis was performed separately for ACS administration to birth intervals of 0 to 24 hours, 0 to 14 days, and 0 to 5 weeks from the first dose. Subgroup analyses were conducted by gestational age at ACS exposure (≤28 weeks of gestation vs >28 weeks of gestation) and by plurality (singletons vs twins). Differences were considered statistically significant for a 2-sided *P* < .05. Data were analyzed from November 29, 2023, to March 8, 2024, using the statistical package SAS version 9.3 (SAS Institute Inc).

## Results

### Characteristics of the Study Cohort

A total of 13 573 neonates between 23 weeks 0 days of gestation and 31 weeks 6 days of gestation were admitted to the CNN/CPTBN during the study period. Of these neonates, 7950 met the study criteria: 1220 (15%) were not exposed to ACS, 2270 (29%) were exposed to ACS less than 24 hours before birth, 2506 (32%) were exposed within 24 hours to 7 days before birth, 861 (11%) were exposed between 8 and 14 days, and 1093 (14%) were exposed more than 14 days before birth (eFigure 1 in Supplement 1).

The baseline characteristics are presented in Table 1. Mean (SD) maternal age was 31.1 (5.7) years. Compared with maternal exposure to ACS, individuals not exposed to ACS were slightly younger, had lower rates of nulliparity, twin pregnancy, hypertensive complications, gestational diabetes, magnesium sulfate prophylaxis, and cesarean delivery and higher rates of outborn status (Table 1). Gestational age at birth was overall similar between the study groups. Neonates not exposed to ACS and those exposed to ACS less than 24 hours before birth had higher mean birth weights than the other groups (Table 1). The mean gestational age at exposure to ACS ranged between 26 and 28 weeks. The overall rates of neonatal mortality before discharge and the composite outcome of neonatal mortality or severe neurologic injury were 8% (670 of 7950) and 14% (1132 of 7950), respectively.

**Table 1.** Characteristics and Outcomes of the Study Cohort

Variable	Neonates or pregnancies, No./total No. (%)^a^						
	No ACS (n = 1220 neonates, 1124 pregnancies)	ACS <24 h (n = 2270 neonates, 2039 pregnancies)	ACS from 24 h to 7 d (n = 2506 neonates, 2251 pregnancies )	ACS from 8 to 14 d (n = 861 neonates, 772 pregnancies)	ACS >14 d (n = 1093 neonates, 938 pregnancies)	*P* value
Maternal age, mean (SD), y	30.3 (6.1)	30.8 (5.6)	31.3 (5.6)	31.4 (5.7)	32.1 (5.4)	<.001
Maternal age >35 y	227/1103 (21)	427/2015 (21)	524/2224 (24)	193/764 (25)	258/930 (28)	<.001
Nulliparity	368/1022 (36)	931/1944 (48)	1033/2067 (50)	317/697 (45)	314/792 (40)	<.001
Twin pregnancy	129/1124 (11)	331/2039 (16)	345/2251 (15)	122/772 (16)	211/938 (22)	<.001
Hypertensive complications^b^	117/1007 (12)	372/2005 (19)	639/2241 (29)	199/771 (26)	166/933 (18)	<.001
Gestational diabetes	118/992 (12)	287/1980 (14)	333/2198 (15)	128/757 (17)	234/925 (25)	<.001
Preterm prelabor rupture of membranes	77/928 (8)	158/1955 (8)	815/2193 (37)	358/747 (48)	416/917 (45)	<.001
Cesarean delivery	636/1220 (52)	1448/2270 (64)	1594/2506 (64)	591/860 (69)	742/1093 (68)	<.001
Outborn	636/1219 (52)	399/2270 (18)	36/2506 (1)	9/861 (1)	17/1093 (2)	<.001
Magnesium sulfate treatment	271/1103 (25)	1777/2213 (80)	2165/2476 (87)	741/846 (88)	921/1077 (86)	<.001
Gestational age at exposure to ACS, mean (SD), wk	NA	28.0 (2.5)	27.8 (2.5)	26.8 (2.3)	25.6 (2.0)	<.001
Gestational age at exposure ≤28 wk	NA	1173/2270 (52)	1345/2506 (54)	598/861 (69)	1008/1093 (92)	<.001
Gestational age at birth, mean (SD), wk	28.0 (2.5)	28.0 (2.5)	27.8 (2.5)	27.8 (2.3)	28.7 (1.9)	<.001
Neonate sex						
Male	725/1220 (59)	1282/2270 (56)	1340/2503 (53)	467/861 (54)	613/1093 (56)	.005
Female	495/1220 (41)	988/2270 (44)	1166/2503 (47)	394/861 (46)	480/1093 (44)	
Birth weight, mean (SD), g	1247 (425)	1213 (415)	1137 (405)	1112 (389)	1264 (358)	<.001
Birth weight <10th percentile	61/1219 (5)	177/2270 (8)	270/2503 (11)	112/861 (13)	108/1093 (10)	<.001
Length of hospital stay, median (IQR), d	42 (17-71)	44 (22-78)	51 (26-87)	53 (30-90)	43 (19-71)	<.001

Abbreviations: ACS, antenatal corticosteroid; NA, not applicable.

^a^ Analyses for neonates use the number of neonates; for maternal characteristics, the unit of analysis is the number of pregnancies. Total numbers vary because of missing data.

^b^ Refers to gestational hypertension or preeclampsia (including HELLP syndrome [hemolysis, elevated liver enzymes, and low platelet count] and eclampsia).

### ACS Exposure to Birth Interval as a Categorical Variable

To facilitate comparison with previously published data, we first estimated the association between the interval from ACS administration to birth as a categorical variable (using categories commonly applied in prior studies) and the study outcomes. Exposure to ACS was associated with a reduction in the risk of the study outcomes irrespective of the interval group (Table 2). The reduction was greatest when ACS were administered between 24 hours and 7 days before birth or between 8 and 14 days before birth (adjusted RR [ARR], 0.50 [95% CI, 0.40-0.63] and 0.51 [95% CI, 0.37-0.39], respectively, for neonatal mortality) and smallest when the exposure occurred less than 24 hours before birth (ARR, 0.75 [95% CI, 0.61-0.91] for neonatal mortality).

**Table 2.** Association of the ACS Administration to Birth Interval Group With the Study Outcomes

Outcome	ACS administration to birth interval												
	None (reference group)	<24 h (n = 2270)			24 h to 7 d (n = 2506)			8-14 d (n = 861)			>14 d (n = 1093)		
	No. (%) (n = 1220)	No. (%)	Crude RR (95% CI)	Adjusted RR (95% CI)^a^	No. (%)	Crude RR (95% CI)	Adjusted RR (95% CI)^a^	No. (%)	Crude RR (95% CI)	Adjusted RR (95% CI)^a^	No. (%)	Crude RR (95% CI)	Adjusted RR (95% CI)^a^
Neonatal mortality	152 (12)	229 (10)	0.81 (0.67-0.99)	0.75 (0.61-0.91)	182 (7)	0.59 (0.48-0.72)	0.50 (0.40-0.63)	55 (6)	0.52 (0.38-0.70)	0.51 (0.37-0.69)	52 (5)	0.39 (0.29-0.53)	0.66 (0.47-0.92)
Neonatal mortality or severe neurologic injury	247 (20)	399 (18)	0.87 (0.75-1.01)	0.82 (0.71-0.94)	279 (11)	0.55 (0.47-0.64)	0.49 (0.42-0.59)	104 (12)	0.60 (0.48-0.74)	0.58 (0.47-0.72)	103 (9)	0.48 (0.38-0.59)	0.68 (0.54-0.86)

Abbreviations: ACS, antenatal corticosteroid; RR, risk ratio.

^a^ Generalized linear models, assuming a Poisson distribution, were used to estimate the RRs with 95 CIs using the nonexposed group as the reference. Twin pairs were considered clusters to account for correlation within the pairs. Models were adjusted for maternal age, hypertensive complications, twin gestation, birth weight below the 10th percentile for gestational age, outborn status, and gestational age at birth.

### ACS Exposure to Birth Interval as a Continuous Variable

**Figure 1.**
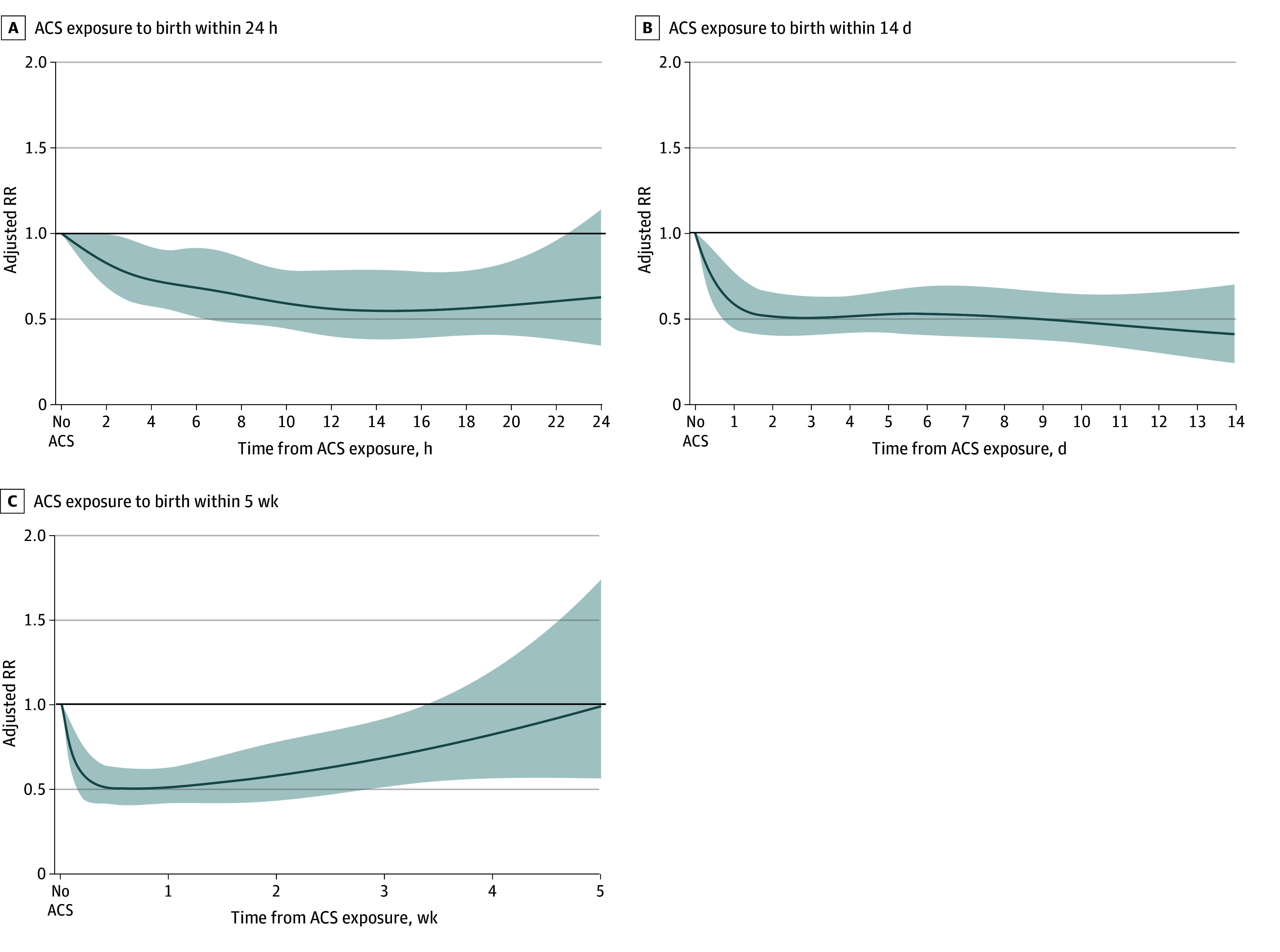
Association of Antenatal Corticosteroid (ACS) Administration to Birth Interval With Neonatal Mortality Associations within the first 24 hours, 14 days, and 5 weeks following administration of the first ACS dose. Lines represent adjusted risk ratios (RRs) with 95% CIs (shading), using neonates not exposed to ACS (represented by interval 0) as the reference group.

**Figure 2.**
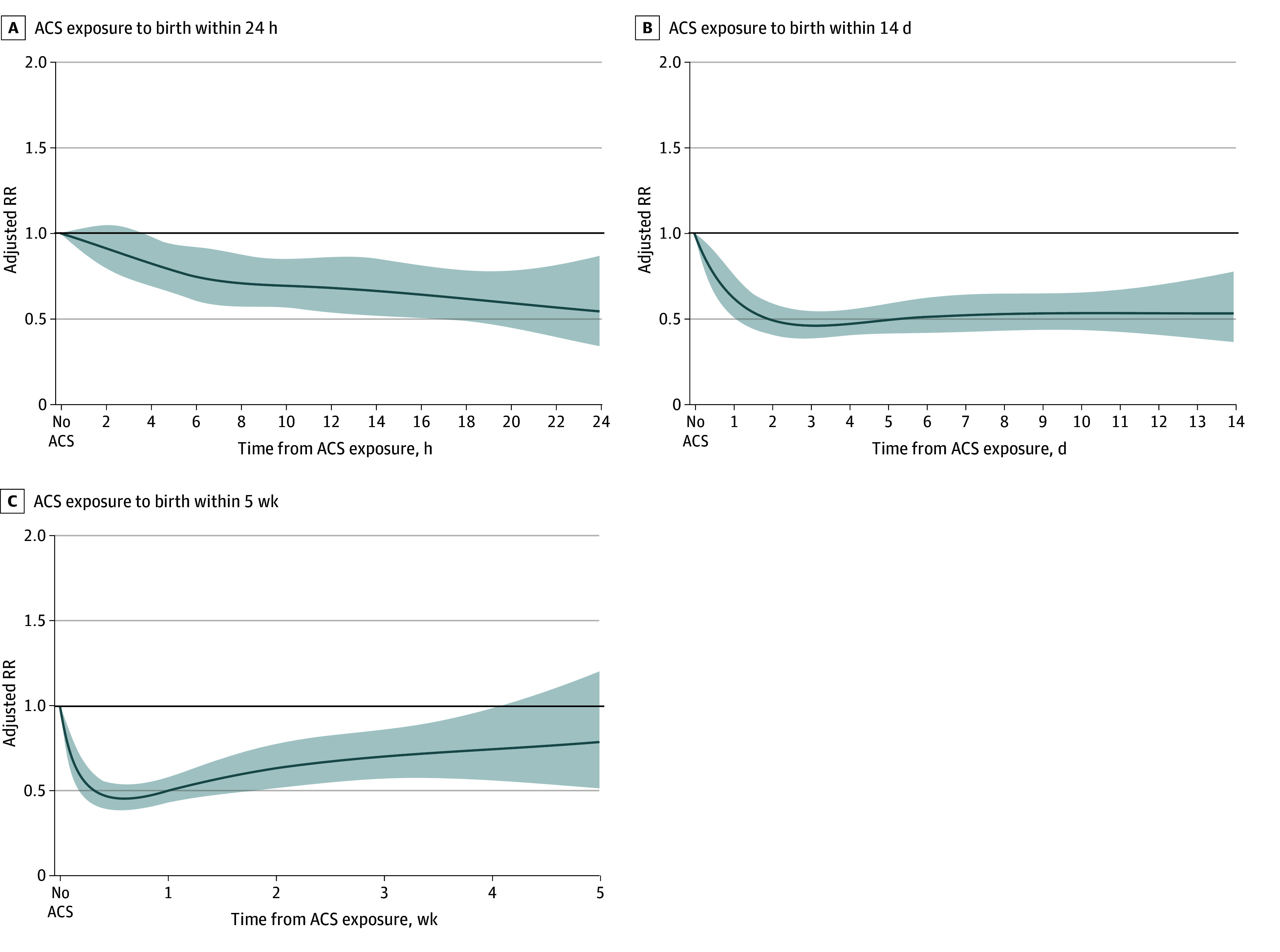
Association of the Antenatal Corticosteroid (ACS) Administration to Birth interval With a Composite Outcome of Mortality or Severe Neurologic Injury Associations within the first 24 hours, 14 days, and 5 weeks following administration of the first ACS dose. Lines represent adjusted risk ratios (RRs) with 95% CIs (shading), using neonates not exposed to ACS (represented by interval 0) as the reference group.

Next, we estimated the association between the interval from ACS administration to birth, treated as a continuous variable, and the study outcomes to identify the shortest interval at which the association becomes apparent and the longest interval at which it persists. ACS administration was associated with a reduction in neonatal mortality as early as 2 hours after administration of the first dose (ARR, 0.83 [95% CI, 0.70-1.00]). The reduction in risk of neonatal mortality associated with ACS use increased, reaching a plateau 12 hours following exposure (ARR, 0.56 [95% CI, 0.40-0.78]) (Figure 1A; eTable 1 in Supplement 1). The reduction in the risk of neonatal mortality remained relatively stable for the first 2 weeks following exposure (Figure 1B) and gradually decreased thereafter. The association was no longer observed 4 weeks after the administration of the first dose (ARR, 0.82 [95% CI, 0.56-1.20]), and the point estimate of the RR approached the null at 5 weeks after administration (ARR, 0.99 [95% CI 0.56-1.73]) (Figure 1C; eTable 1 in Supplement 1). This pattern of findings was generally similar for the secondary composite outcome of neonatal mortality or severe neurologic injury (Figure 2; eTable 2 in Supplement 1). The association of ACS with outcomes over time was relatively similar for neonates born at 28 or less weeks of gestation vs longer than 28 weeks of gestation (eFigure 2 in Supplement 1) as well as for singleton vs twin neonates (eFigure 3 in Supplement 1).

## Discussion

In this large, population-based, national cohort study of preterm neonates born before 32 weeks of gestation, we identified 3 key findings. First, the administration of ACS was associated with a reduction in neonatal mortality and morbidity as early as 2 hours after the first dose, with this association remaining statistically significant until 4 weeks after the administration. Second, the maximal reduction (of approximately 50%) in neonatal mortality and morbidity was observed between 12 hours and approximately 14 days after the first ACS dose. Finally, this pattern of association between the interval of ACS administration to birth and study outcomes was consistently observed among neonates both before and after 28 weeks of gestation, as well as among singleton and twin neonates.

Precise information on the association between the timing of ACS exposure and neonatal mortality and morbidity can help guide management decisions, such as whether ACS should be administered in cases in which preterm birth is imminent and expected within the next few hours, identifying the optimal timing of ACS, and guiding the timing of a repeat course of ACS for pregnant individuals who remain pregnant several weeks after the first ACS course.^16,26^ However, data on this association are limited, as most published studies have treated the interval between ACS administration and birth as a categorical variable with wide predetermined time intervals.^27^ For instance, many studies have used ACS administration to birth intervals of less than 24 hours and more than 7 days as the shortest and longest interval categories, respectively, making it impossible to determine the exact onset of the associated outcomes within the first 24 hours and the duration of the associations beyond the first 7 days from administration.^27,36^ By considering the ACS administration to birth interval as a continuous variable, we found that ACS exposure was associated with a reduction in neonatal mortality when administered between 2 hours and 4 weeks before birth, while the maximal reduction in risk of neonatal mortality (optimal interval) was observed within the interval of 12 hours and approximately 14 days before birth, contrasting with the current view of an optimal interval of 1 to 7 days. The mechanisms underlying the benefits associated with ACS exposure within hours of administration are unclear but are supported by pharmacological studies^37^ and likely involve nongenomic pathways of ACS action.^38,39^

**Figure 3.**
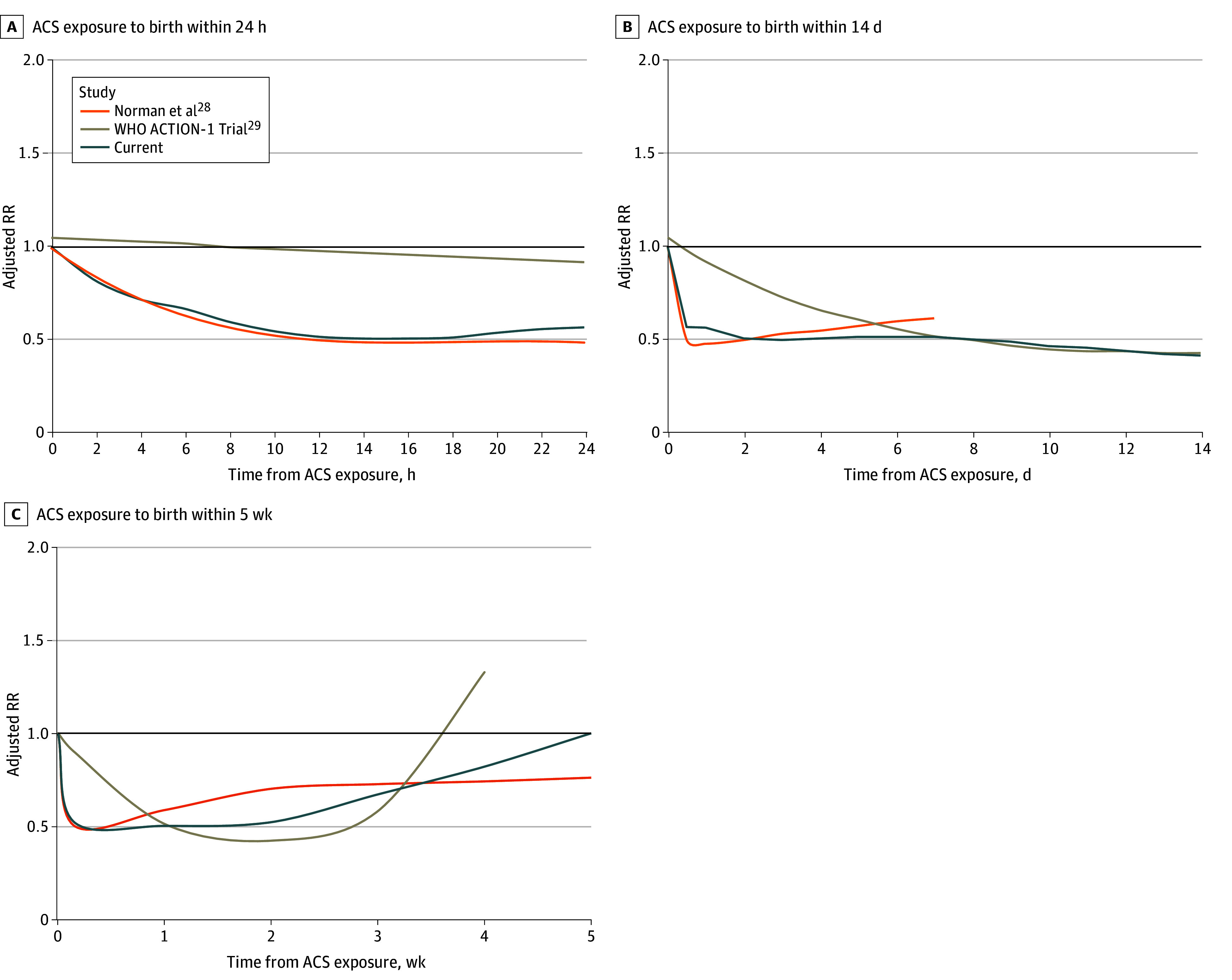
Association of the Antenatal Corticosteroid (ACS) Administration to Birth Interval With Neonatal Mortality Compared With Published Studies Associations within the first 24 hours, 14 days, and 5 weeks following the administration of the first ACS dose observed in the current study with that reported in the study by Normal et al^28^ and a secondary analysis of the WHO ACTION-I trial.^29^ Lines represent the adjusted risk ratio (RR).

Only a small number of previous studies have considered the ACS administration to birth interval as a continuous variable.^28,29^ In an observational study of 4594 singleton infants born between 24 and 31 weeks’ gestation, Norman et al^28^ reported that the association of ACS with a reduction in neonatal mortality was noticed as early as the first few hours following administration. The risk reduction increased rapidly over the first 12 hours, and the association remained significant for up to 3 to 4 weeks following administration. These observations are nearly identical to those of our study (Figure 3). Regarding the optimal interval, they found the association to be more transient than in our study, with a gradual decrease in the reduction in neonatal mortality associated with ACS exposure beginning 5 to 7 days after administration. Thus, the optimal interval in their study was identified as being from 12 hours to 5 to 7 days (Figure 3). In addition, while we found that the risk reduction became statistically nonsignificant at 4 weeks and reached the null at 5 weeks after administration, the point estimate of the RR in the study by Norman et al^28^ remained unchanged between 1 and 5 weeks at approximately 25% risk reduction (Figure 3). The reasons for these differences are unclear. One possible explanation is the smaller number of cases with longer ACS administration to birth intervals, which may affect the accuracy and precision (as reflected by the wider confidence intervals) of the risk estimates at longer intervals. A second potential explanation is that longer ACS administration to birth intervals are associated with more advanced gestational age at birth (ie, milder prematurity), at which time the benefits associated with ACS exposure are known to be less pronounced, particularly in the current era of neonatal care.^6^

In a recent secondary analysis of data from a multicenter randomized clinical trial in low-resource countries (WHO ACTION-I trial^29^), the authors evaluated the effect of dexamethasone vs placebo on newborn mortality and severe respiratory distress at different administration to birth intervals among 2904 infants exposed to dexamethasone or placebo between 26 weeks 0 days of gestation and 33 weeks 6 days of gestation. They found that the association between dexamethasone and a reduction in the study outcomes increased gradually over time, peaked at approximately 14 days, and then diminished as the interval approached 28 days (Figure 3).^29^ The authors concluded that the neonatal benefits of dexamethasone appear to increase with longer administration to birth intervals than previously thought. In contrast to our study and the findings of Norman et al,^28^ they found that dexamethasone was not associated with a reduction in neonatal mortality and morbidity within the first 24 hours after administration of the first dose (Figure 3). Thus, compared with our findings, the beneficial interval of ACS exposure in their study was narrower (3 days to 3-4 weeks), and the optimal interval was shifted to the right (approximately 1-3 weeks) (Figure 3). Possible reasons for these conflicting findings include differences in the study population (the WHO ACTION-I study^29^ included participants from low-resource countries, and nearly two-thirds of the participants had their first dating ultrasonogram during the third trimester), differences in the gestational age at administration of ACS (>90% of the participants in their study were exposed to ACS between 28 and 34 weeks with a mean gestational age at exposure of 31 weeks, as opposed to 23 weeks 0 days to 31 weeks 6 days’ gestation in the current study with mean gestational age at exposure of 26-28 weeks), and differences in the ACS treatment regimen. Another potential explanation for the conflicting results is the statistical approach used to model the association between the ACS administration to birth interval and neonatal outcomes, which can affect this association, especially at extreme (very short or very long) intervals. Unlike the current study and the study of Norman et al,^28^ which used restricted cubic splines, the WHO ACTION-I study^29^ used quadratic polynomial equations, which allows for less flexibility in fitting complex nonlinear patterns.

While acknowledging the limitations inherent to the observational nature of the current study, these findings may have important clinical implications for the management of pregnancies at risk of preterm birth. First, these findings may support the administration of ACS even in cases where preterm birth is imminent and expected within the next few hours. Second, the observation that the association of ACS with reduced neonatal mortality persisted for 3 to 4 weeks may influence the timing or necessity of a repeat (rescue) course of ACS in centers where repeat courses are considered.^16,26^ Third, our finding that the optimal ACS administration to birth interval may extend up to 2 weeks (as opposed to 1 week) from the first dose suggests the need for a reassessment of the current recommendation by nearly all professional societies that ACS should be given when delivery is anticipated within 7 days.^7,16,42,43,44,45,46^ However, additional, large-scale studies are needed to confirm or refute our observations and resolve some of the conflicting findings, particularly in cases with long ACS administration to birth intervals.

### Strengths and Limitations

The main strengths of the current study include the large national cohort and the use of a validated database. The substantial sample size allowed us to adjust the analysis for potential confounders and obtain estimates with high resolution and over a wide range of ACS administration to birth intervals. In addition, the current study addresses an important knowledge gap with regard to the beneficial and optimal ACS administration to birth intervals in relation to the association with neonatal mortality and morbidity.

Our study has limitations. First, this was a retrospective cohort study, and although we controlled for known confounding variables, there remains the possibility for residual confounding due to variables for which information is not available (eg, etiology for preterm birth or reason for failure to administer ACS).^40^ Still, such population-based studies are likely to remain the primary source of information on these questions, as it is methodologically impossible to randomize patients to different ACS administration to birth intervals. Second, although we analyzed a large national cohort, the precision of the estimates for very long ACS administration to birth intervals was relatively low (as reflected by the wide confidence intervals) due to the smaller number of observations at these intervals. Third, we lacked information on the proportion of neonates exposed to betamethasone vs dexamethasone, and we cannot rule out that the association between the timing of administration and outcomes differs between these 2 drugs. However, it is likely that the majority of neonates were exposed to betamethasone, as it is the most commonly used type of ACS in Canada. In addition, a systematic review^41^ found no differences between betamethasone and dexamethasone in terms of neonatal death and intraventricular hemorrhage. Fourth, there is the possibility of left truncation bias as we only studied neonates born before 32 weeks of gestation, while those who had received ACS but were born at 32 weeks and beyond were excluded. Thus, for example, data for ACS administration to birth interval of 5 or more weeks were limited to neonates who were exposed to ACS at or before 27 weeks of gestation. Fifth, we did not consider respiratory distress syndrome as an outcome due to the challenges of ascertaining this diagnosis given the changes in postnatal respiratory practices. Instead, we chose to focus on major and robust clinical outcomes (mortality and severe neurologic morbidity). Additionally, information on maternal race and ethnicity was not available in our database. We believe, on the basis of Canada’s universal health care system, that disparities in health care access and quality of care may be lower than in other settings. However, we fully acknowledge that nonclinical factors related to race and ethnicity (eg, clinician-patient interactions and social determinants of health) may still influence health care delivery. Therefore, we cannot entirely rule out some degree of confounding due to race and ethnicity.

## Conclusions

In the current cohort study, by considering the ACS administration to birth interval as a continuous variable, we refined the association between the timing of ACS administration and the reduction in neonatal mortality and morbidity. We identified that ACS exposure was associated with a reduction in neonatal mortality and morbidity as early as 2 hours after the administration of the first dose. Furthermore, the optimal ACS administration to birth interval associated with the greatest reduction in neonatal mortality was between 12 hours and 14 days, which is wider than the currently accepted optimal interval of 1 to 7 days. These findings may have important clinical implications for practice, including the administration of ACS even when preterm birth is imminent, and the decision and timing of a repeat course of ACS in centers where repeat courses are considered.

## Notes

Supplement 1. **eFigure 1.** Description of the study cohort **eTable 1.** Association of the ACS-to-birth interval with neonatal mortality **eTable 2.** Association of the ACS-to-birth interval with a composite outcome of mortality or severe neurological injury **eFigure 2.** Association of the ACS-to-birth interval with neonatal mortality stratified by gestational age at birth **eFigure 3.** Association of the ACS-to-birth interval with neonatal mortality stratified by plurality

Supplement 2.
Nonauthor Collaborators

Supplement 3.
Data Sharing Statement
