# Supplementary material for: Timing of Antenatal Corticosteroid Administration and Neonatal Outcomes
Source: JAMA Netw Open. 2025 May 19;8(5):e2511315. doi: 10.1001/jamanetworkopen.2025.11315 (PMC12090034; doi:10.1001/jamanetworkopen.2025.11315)
Supplement: Supplement 1. — eFigure 1. Description of the study cohort eTable 1. Association of the ACS-to-birth interval with neonatal mortality eTable 2. Association of the ACS-to-birth interval with a composite outcome of mortality or severe neurological injury eFigure 2. Association of the ACS-to-birth interval with neonatal mortality stratified by gestational age at birth eFigure 3. Association of the ACS-to-birth interval with neonatal mortality stratified by plurality [file jamanetwopen-e2511315-s001.pdf]

Supplementary Online Content

Melamed N, Murphy KE, Pylypjuk C, et al. Timing of antenatal corticosteroid administration and neonatal outcomes. *JAMA Netw Open*. 2025;8(5):e2511315.
doi:10.1001/jamanetworkopen.2025.11315

eFigure 1. Description of the study cohort

eTable 1. Association of the ACS-to-birth interval with neonatal mortality

eTable 2. Association of the ACS-to-birth interval with a composite outcome of mortality or severe neurological injury

eFigure 2. Association of the ACS-to-birth interval with neonatal mortality stratified by gestational age at birth

eFigure 3. Association of the ACS-to-birth interval with neonatal mortality stratified by plurality

This supplementary material has been provided by the authors to give readers additional information about their work.

© 2025 Melamed N et al. *JAMA Network Open*.

eFigure 1. Description of the study cohort

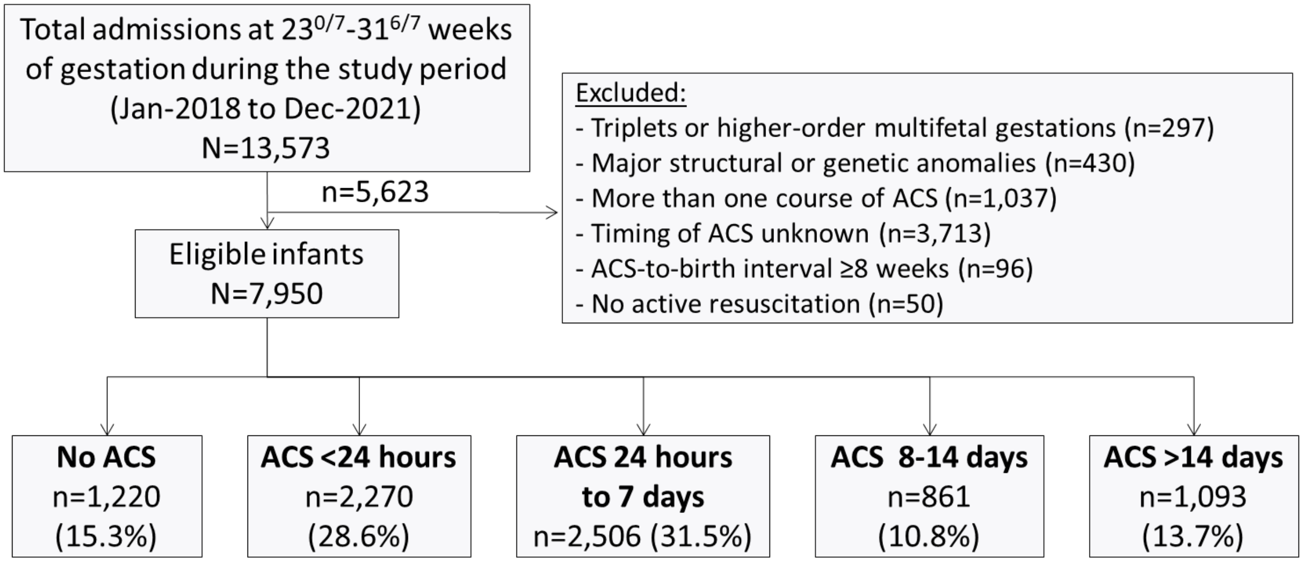

ACS, antenatal corticosteroids

© 2025 Melamed N et al. *JAMA Network Open*.

eTable 1. Association of the ACS-to-birth interval with neonatal mortality

**Interval unit**	**ACS-to-birth interval [a]**	**Adjusted RR [b]**	**95%-CI**
**Hours**	No ACS	Reference	Reference
	1	0.912	0.831-1.000
	2	**0.833**	**0.696-0.997**
	3	**0.759**	**0.600-0.962**
	4	**0.725**	**0.573-0.916**
	5	**0.703**	**0.547-0.903**
	6	**0.685**	**0.512-0.915**
	7	**0.664**	**0.488-0.903**
	8	**0.640**	**0.475-0.863**
	9	**0.611**	**0.461-0.809**
	10	**0.590**	**0.443-0.785**
	11	**0.573**	**0.420-0.781**
	12	**0.560**	**0.400-0.785**
	13	**0.552**	**0.387-0.789**
	14	**0.548**	**0.382-0.788**
	15	**0.548**	**0.384-0.782**
	16	**0.551**	**0.390-0.777**
	17	**0.556**	**0.399-0.775**
	18	**0.563**	**0.406-0.781**
	19	**0.572**	**0.409-0.799**
	20	**0.581**	**0.406-0.833**
	21	**0.594**	**0.394-0.898**
	22	**0.605**	**0.379-0.966**
	23	0.616	0.363-1.046
	24	0.627	0.345-1.140
**Days**	No ACS	Reference	Reference
	1	0.586	**0.442-0.778**
	2	0.511	**0.401-0.651**
	3	0.501	**0.399-0.630**
	4	0.514	**0.418-0.631**
	5	0.523	**0.413-0.663**
	6	0.525	**0.402-0.687**
	7	0.520	**0.392-0.689**
	8	0.509	**0.384-0.675**
	9	0.495	**0.373-0.656**
	10	0.478	**0.356-0.641**
	11	0.458	**0.327-0.640**
	12	0.440	**0.298-0.651**
	13	0.423	**0.267-0.671**
	14	0.407	**0.238-0.697**
**Weeks**	No ACS	Reference	Reference

© 2025 Melamed N et al. *JAMA Network Open*.

	1	**0.512**	**0.418-0.627**
	2	**0.580**	**0.432-0.778**
	3	**0.686**	**0.514-0.916**
	4	0.823	0.564-1.201
	5	0.988	0.563-1.733

ACS, antenatal corticosteroids; RR, risk ratio; CI, confidence interval.
[a] Refers to the interval following the administration of the first dose.
[b] The adjusted RR were calculated using infants not exposed to ACS as the reference group. Models were adjusted for the following variables: maternal age, plurality (twins vs. singletons), hypertensive disorders of pregnancy (preeclampsia and gestational hypertension), birthweight below the 10th percentile for gestational age, outborn admission, and gestational age at birth. Significant associated are emphasized using a bold font.

eTable 2. Association of the ACS-to-birth interval with a composite outcome of mortality or severe neurological injury

**Interval unit**	**ACS-to-birth interval [a]**	**Adjusted RR [b]**	**95%-CI**
**Hours**	No ACS	Reference	Reference
	1	0.958	0.895-1.025
	2	0.917	0.804-1.046
	3	0.865	0.728-1.028
	4	0.821	**0.692-0.973**
	5	0.780	**0.651-0.934**
	6	0.746	**0.606-0.919**
	7	0.723	**0.580-0.902**
	8	0.708	**0.571-0.878**
	9	0.697	**0.570-0.853**
	10	0.692	**0.565-0.848**
	11	0.687	**0.552-0.854**
	12	0.680	**0.538-0.861**
	13	0.673	**0.526-0.861**
	14	0.664	**0.517-0.852**
	15	0.651	**0.509-0.832**
	16	0.640	**0.504-0.812**
	17	0.629	**0.498-0.794**
	18	0.617	**0.488-0.780**
	19	0.605	**0.472-0.775**
	20	0.593	**0.451-0.779**
	21	0.577	**0.419-0.795**
	22	0.566	**0.393-0.815**
	23	0.554	**0.366-0.839**
	24	0.543	**0.340-0.867**
**Days**	No ACS	Reference	Reference
	1	0.628	**0.513-0.768**

© 2025 Melamed N et al. *JAMA Network Open*.

	2	0.493	**0.410-0.592**
	3	0.458	**0.383-0.548**
	4	0.472	**0.404-0.552**
	5	0.493	**0.413-0.588**
	6	0.509	**0.417-0.621**
	7	0.520	**0.422-0.640**
	8	0.527	**0.429-0.646**
	9	0.530	**0.435-0.647**
	10	0.532	**0.434-0.651**
	11	0.532	**0.423-0.669**
	12	0.531	**0.405-0.696**
	13	0.531	**0.385-0.732**
	14	0.531	**0.363-0.775**
**Weeks**	No ACS	Reference	Reference
	1	**0.503**	**0.434-0.583**
	2	**0.635**	**0.518-0.778**
	3	**0.704**	**0.575-0.862**
	4	**0.746**	**0.564-0.987**
	5	0.790	0.517-1.205

ACS, antenatal corticosteroids; RR, risk ratio; CI, confidence interval.
[a] Refers to the interval following the administration of the first dose.
[b] The adjusted RR were calculated using infants not exposed to ACS as the reference group. Models were adjusted for the following variables: maternal age, plurality (twins vs. singletons), hypertensive disorders of pregnancy (preeclampsia and gestational hypertension), birthweight below the 10th percentile for gestational age, outborn admission, and gestational age at birth. Significant associated are emphasized using a bold font.

© 2025 Melamed N et al. *JAMA Network Open*.

eFigure 2. Association of the ACS-to-birth interval with neonatal mortality stratified by gestational age at birth

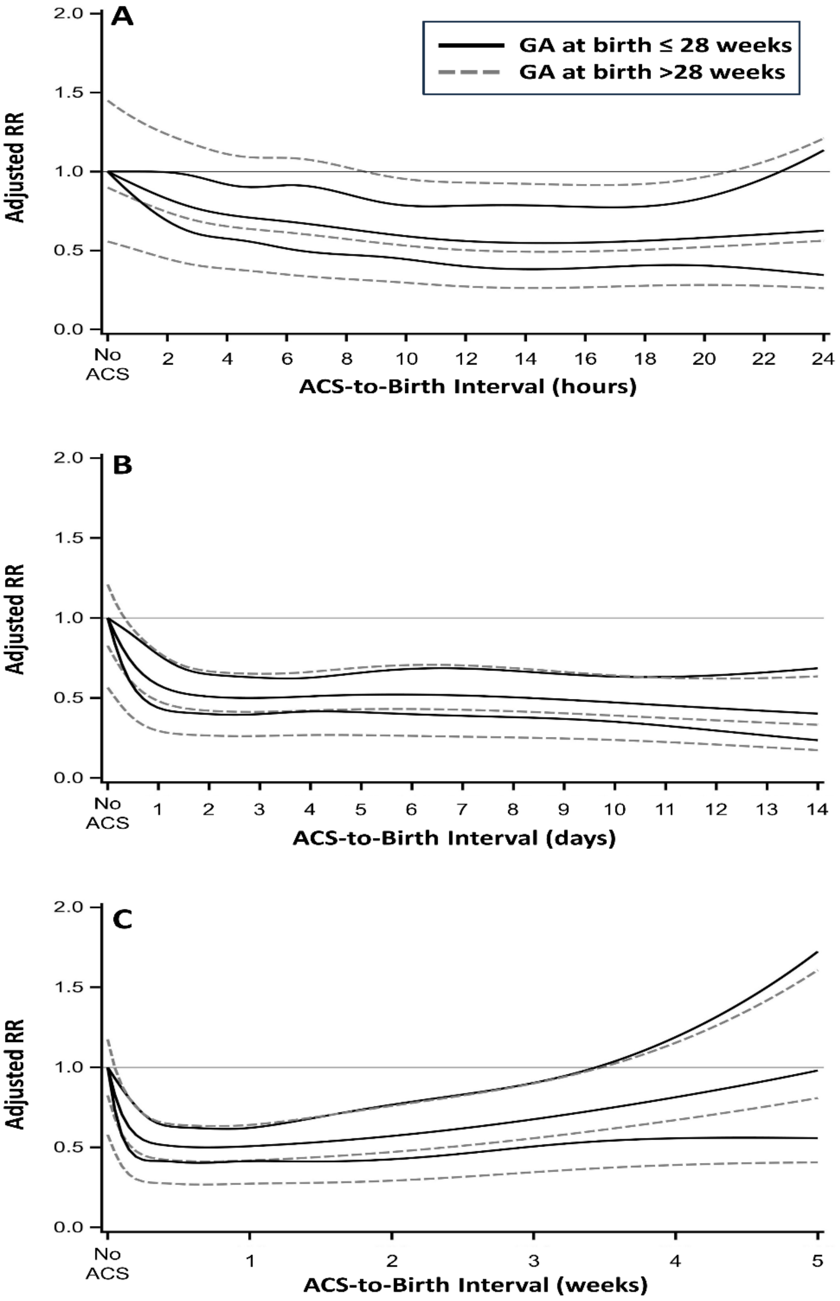

The graph presents the association of neonatal mortality with the ACS-to-birth interval for infants born at ≤28 weeks (solid line) vs. >28 weeks (dashed line). Data are presented for ACS-to-birth interval within the first 24 hours (A), 14 days (B), and 5 weeks (C) following the administration of the first dose. Lines represent the adjusted RR with 95%-CI using infants not exposed to ACS as the reference group.
ACS, antenatal corticosteroids; RR, risk ratio; CI, confidence interval.

© 2025 Melamed N et al. *JAMA Network Open*.

eFigure 3. Association of the ACS-to-birth interval with neonatal mortality stratified by plurality

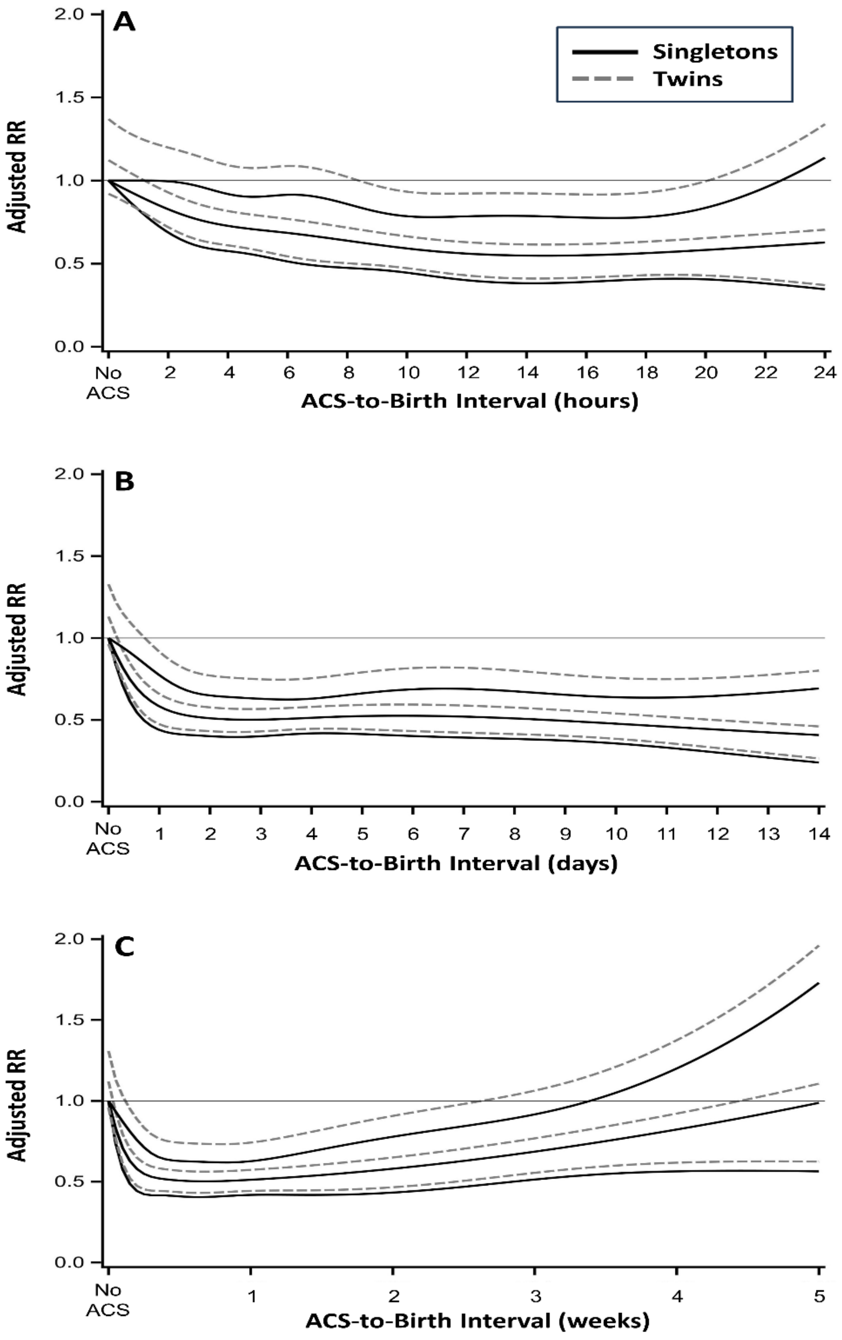

The graph presents the association of neonatal mortality with the ACS-to-birth interval for singleton (solid line) vs. twin (dashed line) infants. Data are presented for ACS-to-birth interval within the first 24 hours (A), 14 days (B), and 5 weeks (C) following the administration of the first dose. Lines represent the adjusted RR with 95%-CI using infants not exposed to ACS as the reference group.
ACS, antenatal corticosteroids; RR, risk ratio; CI, confidence interval.

© 2025 Melamed N et al. *JAMA Network Open*.
