# Supplementary material for: Timing of Antenatal Corticosteroid Administration and Neonatal Outcomes
Source: JAMA Netw Open. 2025 May 19;8(5):e2511315. doi: 10.1001/jamanetworkopen.2025.11315 (PMC12090034; doi:10.1001/jamanetworkopen.2025.11315)
Supplement: Supplement 2. — Nonauthor Collaborators [file jamanetwopen-e2511315-s002.pdf]

Supplemental Online Content: Nonauthor Collaborators
*First name, last name, and suffix (if applicable) are required and will appear in PubMed.

*Group Name(s): the Canadian Neonatal Network (CNN) and Canadian Preterm Birth Network (CPTBN) Investigators							
***First Name and Middle Initial(s)**	***Last Name**	***Suffix (eg, Jr, III)**	Academic Degrees	Institution	Location (city, state/province, country)	Role or Contribution, eg, chair, principal investigator	Group (if more than 1 Group listed in the byline) and/or Subgroup (eg, Steering Committee)
Carlos	Fajardo		MD	Alberta Children's Hospital	Calgary, AB	CNN Site Investigator	
Jonathan	Wong		BSc, MD, MScCH	British Columbia Women's Hospital	Vancouver, BC	CNN Site Investigator	
Andrzej	Kajetanowicz		MD	Cape Breton Regional Hospital	Sydney, NS	CNN Site Investigator	
Bruno	Piedboeuf		MD, FRCPC	Centre Hospitalier Universitaire de Quebec	Sainte Foy, QC	CNN Co-Site Investigator	
Christine	Drolet		MD	Centre Hospitalier Universitaire de Quebec	Sainte Foy, QC	CNN Co-Site Investigator	
Valerie	Bertelle		MD	Centre Hospitalier Universitaire de Sherbrooke	Fleurimont, QC	CNN Co-Site Investigator	
Edith	Masse		MD	Centre Hospitalier Universitaire de Sherbrooke	Fleurimont, QC	CNN Co-Site Investigator	
Anie	Lapointe		MD	Centre Hospitalier Universitaire Sainte-Justine	Montreal, QC	CNN Co-Site Investigator	
Guillaume	Ethier		NNP	Centre Hospitalier Universitaire Sainte-Justine	Montreal, QC	CNN Co-Site Investigator	
Keith	Barrington		MBChB	Centre Hospitalier Universitaire Sainte-Justine	Montreal, QC	CNN Co-Site Investigator	
Brigitte	Lemyre		MD	Children's Hospital of Eastern Ontario	Ottawa, ON	CNN Site Investigator	
Hala	Makary		MD	Dr. Everett Chalmers Hospital	Fredericton, NB	CNN Co-Site Investigator	
Ahmad	Aziz		MD	Dr. Everett Chalmers Hospital	Fredericton, NB	CNN Co-Site Investigator	
Ayman	Abou Mehrem		MD, MSc	Foothills Medical Centre	Calgary, AB	CNN Site Investigator	
Amit	Mukerji		MD, MSc, FRCPC	Hamilton Health Sciences Centre	Hamilton, ON	CNN Site Investigator	
Mary	Seshia		MBChB	Health Sciences Centre	Winnipeg, MB	CNN Co-Site Investigator	
Deepak	Louis		MD	Health Sciences Centre	Winnipeg, MB	CNN Co-Site Investigator	
Kyong-Soon	Lee		MD, MSc	Hospital for Sick Children	Toronto, ON	CNN Site Investigator	
Jehier	Afifi		MBChB, MSc	Izzak Walton Killam (IWK) Health Centre	Halifax, NS	CNN Site Investigator	
Jo-Anna	Hudson		BSc, PhD, MD, FRCPC	Janeway Children's Health and Rehabilitation Centre	St. Johns, NL	CNN Site Investigator	
Victoria	Bizgu		MD	Jewish General Hospital	Montreal, QC	CNN Co-Site Investigator	
Nina	Nouraeyen		MD	Jewish General Hospital	Montreal, QC	CNN Co-Site Investigator	
Faiza	Khurshid		MD	Kingston Health Sciences Centre	Kingston, ON	CNN Site Investigator	

Supplemental Online Content: Nonauthor Collaborators
*First name, last name, and suffix (if applicable) are required and will appear in PubMed.

*First Name and Middle Initial(s)	*Last Name	*Suffix (eg, Jr, III)	Academic Degrees	Institution	Location (city, state/province, country)	Role or Contribution, eg, chair, principal investigator	Group (if more than 1 Group listed in the byline) and/or Subgroup (eg, Steering Committee)
Kevin	Coughlin		BScH, MD, MHSc, FRCPC	London Health Sciences Centre	London, ON	CNN Site Investigator	
Marie	St-Hilaire		MD	Maisonneuve-Rosemont Hospital	Montreal, QC	CNN Site Investigator	
Marc	Beltempo		MD, MSc, FRCPC	McGill University Health Centre	Montreal, QC	CNN Director and Co-site Investigator	
Marco	Zeid		NNP, MSc(N)	McGill University Health Centre	Montreal, QC	CNN Co-Site Investigator	
Prakeshkumar	Shah		MD, FRCPC, MRCP, MSc	Mount Sinai Hospital	Toronto, ON	CNN Site Investigator	
Andrei	Harabor		MD, MSc	Regina General Hospital	Regina, SK	CNN Site Investigator	
Jennifer	Toye		MD	Royal Alexandra Hospital & University of Alberta Hospital	Edmonton, AB	CNN Co-Site Investigator	
Joseph	Ting		MD, FRCPC, MPH	Royal Alexandra Hospital & University of Alberta Hospital	Edmonton, AB	CNN Co-Site Investigator	
Miroslav	Stavel		MD	Royal Columbian Hospital	New Westminster, BC	CNN Site Investigator	
Lannae	Strueby		MD, FRCPC	Royal University Hospital / Jim Pattison Children's Hospital	Saskatoon, SK	CNN Site Investigator	
Gabriela	Nunes		MD	Saint John Regional Hospital	St. John, NB	CNN Co-Site Investigator	
Wissam	Alburaki		MD	Saint John Regional Hospital	St. John, NB	CNN Co-Site Investigator	
Ann	Yi		MD	St. Boniface General Hospital	Winnipeg, MB	CNN Co-Site Investigator	
Chelsea	Ruth		MD, MSc, FRCPC	St. Boniface General Hospital	Winnipeg, MB	CNN Co-Site Investigator	
Eugene	Ng		MD, FRCPC, FAAP,	Sunnybrook Health Sciences Centre	Toronto, ON	CNN Site Investigator	
Rebecca	Sherlock		MD	Surrey Memorial Hospital	Surrey, BC	CNN Site Investigator	
Paloma	Costa		MD	The Moncton Hospital	Moncton, NB	CNN Site Investigator	
Brigitte	Lemyre		MD	The Ottawa Hospital	Ottawa, ON	CNN Site Investigator	
Thevanisha	Pillay		MBChB, FRCPC	Victoria General Hospital	Victoria, BC	CNN Site Investigator	
Sajit	Augustine		MD	Windsor Regional Hospital	Windsor, ON	CNN Site Investigator	
