# Supplementary material for: Timing of Antenatal Corticosteroid Administration and Neonatal Outcomes
Source: JAMA Netw Open. 2025 May 19;8(5):e2511315. doi: 10.1001/jamanetworkopen.2025.11315 (PMC12090034; doi:10.1001/jamanetworkopen.2025.11315)
Supplement: Supplement 3. — Data Sharing Statement [file jamanetwopen-e2511315-s003.pdf]

Data Sharing Statement

Melamed. Timing of Antenatal Corticosteroid Administration and Neonatal Outcomes. *JAMA Netw Open*. Published May 19, 2025. doi:10.1001/jamanetworkopen.2025.11315

Data
Data available: No

Additional Information
Explanation for why data not available: Sharing of data is not permitted per the CNN policy
